# Effects of Different Types of Jump Impact on Trabecular Bone Mass and Microarchitecture in Growing Rats

**DOI:** 10.1371/journal.pone.0107953

**Published:** 2014-09-18

**Authors:** Yong-In Ju, Teruki Sone, Kazuhiro Ohnaru, Kensuke Tanaka, Hidetaka Yamaguchi, Masao Fukunaga

**Affiliations:** 1 Department of Health and Sports Sciences, Kawasaki University of Medical Welfare, Kurashiki, Okayama, Japan; 2 Department of Nuclear Medicine, Kawasaki Medical School, Kurashiki, Okayama, Japan; 3 Department of Orthopedic Surgery, Kawasaki Medical School, Kurashiki, Okayama, Japan; 4 Department of Sports Social Management, Sports Health Course School of Social Science, KIBI International University, Takahashi, Okayama, Japan; 5 Kawasaki Medical School, Kurashiki, Okayama, Japan; Université Jean Monnet, France

## Abstract

Substantial evidence from animal studies indicates that jumping increases bone mass and strength. However, most studies have focused on the take-off, rather than the landing phase of jumps. Thus, we compared the effects of landing and upward jump impact on trabecular bone mass and microarchitecture. Male Wistar rats aged 10 weeks were randomly assigned to the following groups: sedentary control (CON), 40-cm upward jumps (40UJ); 40-cm drop jumps (40DJ); and 60-cm drop jumps (60DJ) (n = 10 each). The upward jump protocol comprised 10 upward jumps/day, 5 days/week for 8 weeks to a height of 40 cm. The drop jump protocol comprised dropping rats from a height of 40 or 60 cm at the same frequency and time period as the 40UJ group. Trabecular bone mass, architecture, and mineralization at the distal femoral metaphysis were evaluated using microcomputed tomography. Ground reaction force (GRF) was measured using a force platform. Bone mass was significantly higher in the 40UJ group compared with the DJ groups (+49.1% and +28.3%, respectively), although peak GRF (−57.8% and −122.7%, respectively) and unit time force (−21.6% and −36.2%, respectively) were significantly lower in the 40UJ group. These results showed that trabecular bone mass in growing rats is increased more effectively by the take-off than by the landing phases of jumps and suggest that mechanical stress accompanied by muscle contraction would be more important than GRF as an osteogenic stimulus. However, the relevance of these findings to human bone physiology is unclear and requires further study.

## Introduction

Although exercise is considered one of the best strategies for enhancing bone mass and strength, not all types of exercise are beneficial. Numerous studies over the last few decades have investigated which types of exercise are more effective for increasing bone mass and strength. High-impact and weight-bearing activity are generally accepted as being beneficial for strengthening skeletal bone. Among various types of exercise in rats, high-impact loading such as jumping seems to be most beneficial for increasing bone mass and strength rather than low-impact loading such as running [1], [2], [3].

The impact transmitted to hindlimb bone during jumps can be divided into take-off and landing phases. Peak ground reaction force (GRF) is generally considered greater during the landing, than the take-off phase [4], [5], [6]. Thus, landing impact is considered more beneficial for building bone mass and increasing strength than take-off impact. The effects of landing (free-fall landing) on bone mass and strength have been investigated in growing rats [7], [8], [9]. Welch et al. [9] found that free-fall landing produced significantly larger increases in strength, geometry, and density in the forelimbs of growing rats. However, upward jump exercise in the form of vertical jumps from the bottom to the top of a board, without GRF at landing has been most widely applied as an exercise model for promoting bone mass, strength, and structure in rodents [10], [11], [12], [13], [14]. Welch et al. [9] also showed that free-fall landing imparts greater osteogenic effects to the bones of the forelimb than the hindlimb in rats [1]. Their report suggested that the type of landing could increase bone mass more effectively than take-off, but the effects of the two jump phases have not been directly compared in the same bone.

We postulated that GRF would be higher at the landing, than at the take-off phase of jumps and that bone mass and quality in the exercised limb would be more effectively improved by the landing, than the take-off impact. We tested this hypothesis by comparing the effects of upward and drop jumps on trabecular bone mass and the microarchitecture of the distal femur in growing rats.

## Materials and Methods

### Ethics statement

This study proceeded in strict accordance with the recommendations described in the Guide for the Care and Use of Laboratory Animals of the National Institutes of Health. The Committee for the Ethics of Animal Experiments at Kawasaki University of Medical Welfare approved the experimental protocol (Permit Number: 10-010). The rats were anesthetized with intraperitoneal pentobarbital sodium (0.1 mL/100 g of body weight) and sacrificed by exsanguination from the abdominal aorta at the end of the experiment.

### Animals and experimental design

Nine-week old male Wistar rats (CLEA Japan, Osaka, Japan), weighing 200–250 g at the start of the experiment, were singly housed in 20×33×14-cm cages in a sanitary ventilated animal room under a controlled temperature (22°C±1°C) and a 12 h/12 h light-dark cycle. The rats were acclimated to the environment, and were given a diet of standard MF laboratory rodent chow (Oriental Yeast Co. Ltd., Chiba, Japan) and water ad libitum for one week before starting the experiment. The rats were weighed and food intake was measured daily before exercise. After one week of acclimation to the diet and new environment, the rats were randomly assigned to the following groups (n = 10 per group): sedentary controls reared in the breeding cage (CON), rats that jumped upwards to a height of 40 cm (40UJ), and rats that fell from a height of 40 (40DJ) or 60 (60DJ) cm. After sacrifice, the right soleus and gastrocnemius muscles were collected from each rat and immediately weighed. Excised femora from each rat were cleaned of soft tissue and then the femoral length was measured using digital calipers. Right femora were stored at −40°C until analysis using micro-computed tomography (micro-CT) and left femora were fixed in 70% ethanol for histomorphometric analysis.

### Exercise conditions

**Figure 1:**
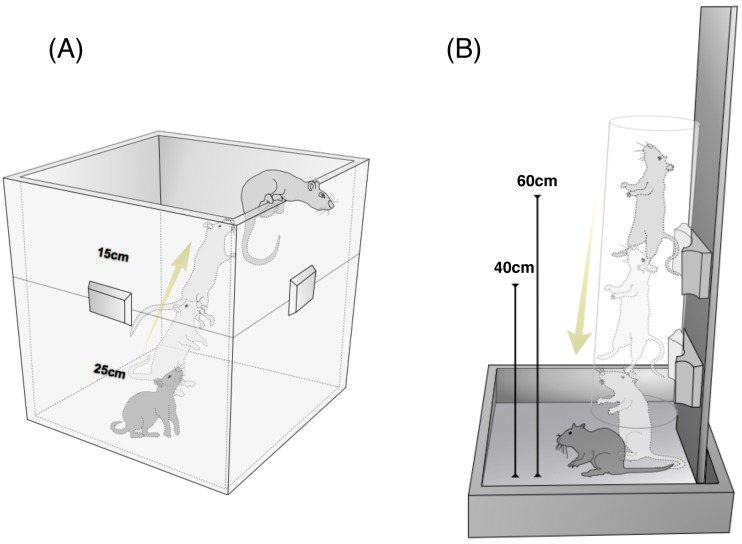
Schematic representation of upward (A) and drop (B) jumps. (A) Rats jumped vertically upward 40 cm from the bottom to the top of a box 10 times per day without landing. (B) Rats landed after being dropped 10 times per day through a 40- or 60-cm-high transparent plastic cylinder without take-off.

Upward jumps proceeded according to our previous publications [11], [12], [13]. Briefly, rats in the 40UJ group were placed individually at the bottom of a wooden box (Figure 1A) and then an electric stimulus was initially provided to force them to jump and grasp the top of one of the sides of the box with the forepaws, climb up onto it and remain there for 11 seconds. A technician then gently placed each rat back on the floor of the box to repeat the procedure without generating GRF above the body weight at landing. Since the rats rapidly became conditioned to jump voluntarily, the electrical stimulus was not required after the first few days. The rats jumped upwards 10 times daily at a frequency of once per 11 seconds, 5 days per week for 8 weeks. The initial height of the box was 25 cm and this was progressively increased to 40 cm during the first week.

Drop jumps proceeded as described with slight modification [9]. In brief, rats were dropped through the inside of a transparent plastic cylinder (internal diameter: 14.5 cm, outside diameter: 15.0 cm) that formed part of a device designed in-house (Figure 1B). The device was fashioned such that the rats would land hindlimb-first on the ground. A preliminary experiment confirmed that the rats indeed landed mostly with hindlimb first when dropped through this device. Rats in the 40DJ and 60DJ groups were dropped 10 times daily onto a bare floor from a height of 40 and 60 cm at the same frequency and duration as the 40UJ group. The 40-cm drop height was selected for comparison with the 40UJ group. Since the 40UJ group grasped the top of the board set at 40 cm with their forelimbs and then climbed onto it, the position in which the drop started was adjusted so that their forelimbs were located 40 cm above the ground. The 60-cm drop height was selected to exceed the impact produced by the 40DJ group. The initial drop height was 25 cm like the 40UJ jump group and progressively increased to 40 cm and 60 cm during the first week. The rats were monitored and jumps were periodically recorded using a high-speed video camera to verify correct and safe landings.

### Determination of GRF

The GRF during take-off or landing for each jump exercise was measured using a Type 9286A multicomponent force platform with dimensions of 40×60 cm (Kistler, Winterthur, Switzerland). Analog voltage signals from each force plate sensor were recorded at a sampling rate of 1000 Hz and sent to a 9865E1Y28 force plate amplifier (Kistler), where they were converted to digital data using a Powerlab 16/30 ML880 system and analyzed using LabChart 7 software (ADInstruments, Nagoya, Japan). The GRF was measured four weeks after the jump program was started. The upward and drop jump devices were installed above the force platform. Rats in the 40UJ group were placed on the force platform from which they jumped 10 times upward to the top edge of the box. The 40DJ or 60DJ rats were dropped 10 times each from a height of 40 or 60 cm onto the force platform using a drop-jump training device. Impulse, peak GRF, contact time, and impulse per contact time (unit time force) from 10 jumps were averaged and the GRF was normalized by body weight (GRF/body weight). Peak GRF was determined as the highest GRF achieved during take-off and landing for each jump.

### Measurement of 3D architectural indices and mineralization density of cancellous bone

Trabecular bone microarchitecture and mineralization were evaluated using an Ele Scan mini micro-CT system (Nittetsu Elex, Tokyo, Japan) that has an X-ray tube with a microfocus (spot size, 6×8 µm) and a maximum resolution of 4 µm (pixel size) [11], [12], [13]. All specimens were scanned at a source energy of 45 kVp and 90 µA to maximize contrast between bone and soft tissue. A 0.1-mm copper plate served as the X-ray filter. The right femur was positioned 2.8–3.0 mm proximal from the distal end of the femur, including the border between the distal metaphysis and the growth plate. We acquired 300 consecutive 18.11 µm (approximately 5.4 mm)-thick tomographic slices and reconstructed CT images with a pixel size of 18.11 µm in 512×512 matrices.

**Figure 2:**
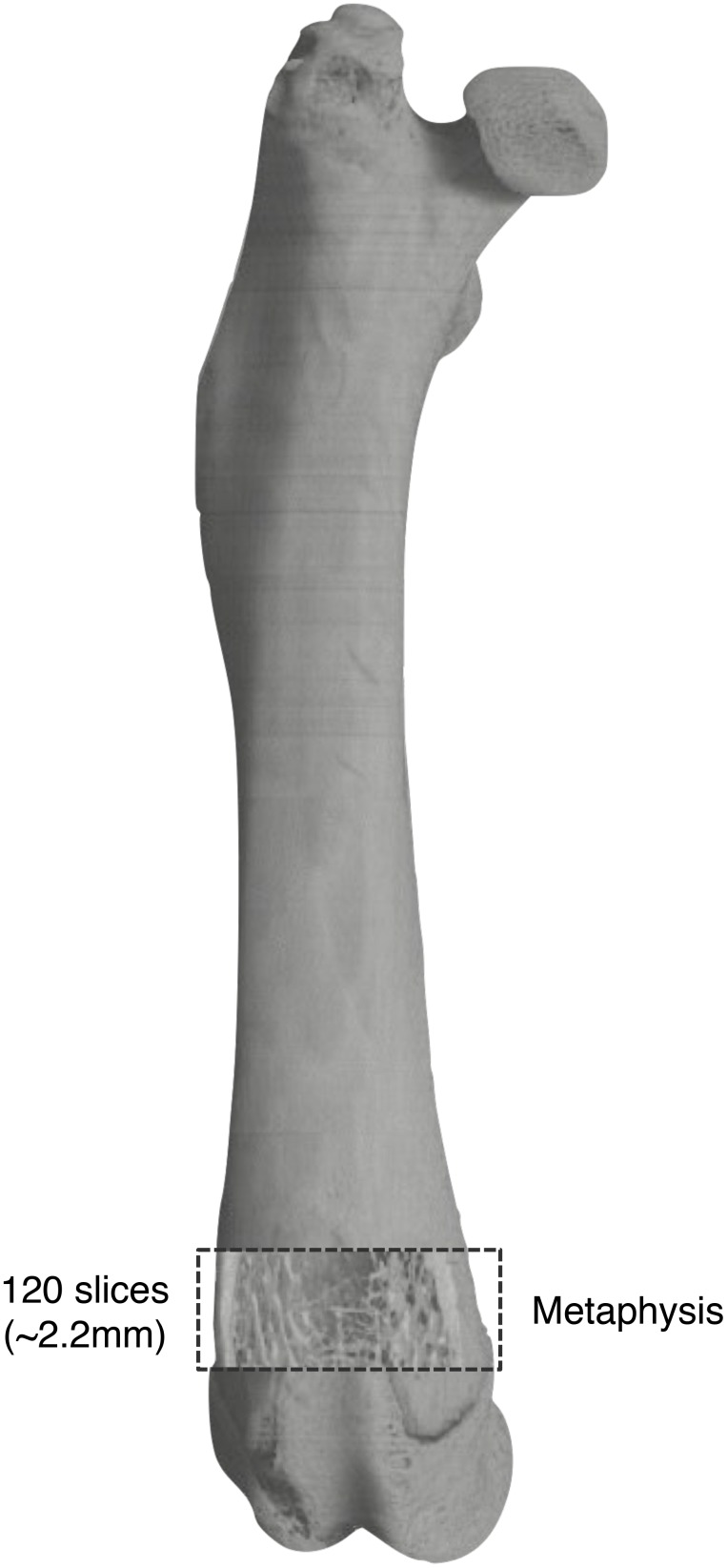
Location of volume of interest in distal femoral metaphysis for micro-CT analysis of trabecular bone.

The microstructural indices and the mineralization density of the trabecular bone were calculated using TRI/3D-BON bone structural analysis software (Ratoc System Engineering, Tokyo, Japan). A volume of interest (VOI) for each femur was defined as 120 slices above the most proximal portion of the growth plate (Figure 2). The resulting gray-scale images were segmented using a 3×3 median filter to remove noise, and a threshold was fixed to extract the mineralized bone phase. Isolated small particles in the marrow space and isolated small holes in bone were removed using a cluster-labeling algorithm. Cortical and trabecular bone were subsequently separated by semi-automatically drawn contours and then structural indices were calculated. The following 3D morphometric parameters were calculated by measuring distances in 3D directly in the trabecular network [15]: total bone marrow volume including trabeculae (TV; mm^3^), trabecular bone volume (BV; mm^3^), trabecular bone volume fraction (BV/TV; %), mean trabecular number (Tb.N;/mm), mean trabecular thickness (Tb.Th; µm), and mean trabecular separation (Tb.Sp; µm). Degrees of trabecular bone mineralization (mineralization density; mg/cm^3^) were measured as described [16]. The CT number was converted into bone mineralization density using a calibration curve obtained from bone mineral density (BMD) measured using a hydroxyapatite phantom (6×1 mm; 200 to 800 mg/cm^3^; Kyoto Kagaku, Kyoto, Japan) and the average degree of mineralization was measured in the region of interest. The BMD phantoms were assessed by CT under the same conditions as actual bone.

### Bone histomorphometry

One and five days before dissection, 8 mg/kg body weight of fluorescent calcein (Dojindo Laboratories, Kumamoto, Japan) in 2% sodium hydrogen carbonate was subcutaneously administered to all rats. The left femur were fixed in 70% ethanol and stained with Villanueva bone stain for six days, dehydrated in graded concentrations of ethanol, and embedded in methyl-methacrylate (Wako Pure Chemical Industries, Osaka, Japan) without bone decalcification. Seven random specimens per group were histomorphometrically analyzed. Fluorochrome-based dynamic histomorphometric parameters were determined in the frontal plane, 5-µm thick sections of the distal metaphysis cut using a RM2255 rotary microtome (Leica, Wetzlar, Germany). A region of interest was positioned 0.9–3.5 mm proximal to the distal femoral growth cartilage/metaphyseal junction, and 0.9 mm from the endosteal border of the cortices. The sections were analyzed at 320× magnification using a semiautomatic image analyzing system (System Supply, Nagano, Japan), linked to a BX-53 light/epifluorescent microscope (Olympus, Tokyo, Japan). Sections were histomorphometrically assessed at the Ito Bone Histomorphometry Institute (Niigata, Japan). The following indices for cancellous bone were determined: BV/TV (%); Tb.Th (µm); Tb.N (/mm); Tb.Sp (µm); osteoclast surface/bone surface (Oc.S/BS, %); eroded surface/BS (ES/BS, %); osteoblast surface/BS (Ob.S/BS, %); mineral apposition rate (MAR, µm/day); mineralizing surface/BS (MS/BS, %); and bone formation rate/BS (BFR/BS, µm^3^/µm^2^/day) [17].

### Statistical analysis

All data were statistically analyzed using the IBM SPSS Statistics 22.0 software (IBM, Armonk, NY, USA). Categories were compared by a one-way analysis of variance followed by Tukey’s post hoc analysis. All data are expressed as means ± SD. The level of statistical significance was set at p<0.05.

## Results

### Body weight, calf muscle weight, and bone length

Table 1 shows body weight before and after the experiment, the weight of the soleus/gastrocnemius (calf) muscles, and the femoral length of the rats in each group. Body weight did not significantly differ throughout the study between the sedentary controls and the three groups of exercised rats. Calf muscles were weighed to confirm the effect of drop and upward jumps on skeletal muscle. The weight of the calf muscles and the length of the femur were similar among the four groups of rats at the end of the experiment. Thus, drop and upward jumps did not affect either skeletal muscle weight or femoral length.

**Table 1:** Body weight, hindlimb muscle weight, and femoral length in experimental rats.

	CON	40DJ	60DJ	40UJ
	(n = 10)	(n = 10)	(n = 10)	(n = 10)
Body weight before experiment (g)	291.79±09.57	292.41±08.63	292.29±17.11	295.21±08.78
Body weight after experiment (g)	421.56±09.68	421.04±22.89	420.01±24.67	420.55±14.88
Calf muscle weight				
Soleus (g)	0.74±0.01	0.73±0.02	0.73±0.01	0.74±0.01
Gastrocnemius (g)	2.48±0.09	2.43±0.20	2.48±0.16	2.47±0.08
Total muscle weight (g)	3.22±0.09	3.16±0.21	3.22±0.17	3.20±0.08
Femoral length (mm)	37.61±0.86	37.46±0.60	37.63±0.68	38.16±0.53

Values are shown as means ± SD. n, number of rats in each group; CON, sedentary control group reared in breeding cage; 40DJ, rats that jumped downwards from a height of 40 cm; 60DJ, rats that jumped downwards from a height of 60 cm; 40UJ, rats that jumped upwards to a height of 40 cm.

### Ground reaction force

Table 2 shows the outcomes of comparisons of peak GRF, impulse, contact time, and unit time force between each group after 4 weeks. Peak GRF, impulse, and unit time force were significantly greater in 40DJ and 60DJ groups than in 40UJ group, except for impulse in the 40DJ group. Peak GRF and unit time force were also significantly greater in the 60DJ, than in the 40DJ group. In contrast, contact time was significantly greater in 40UJ, than in the 40DJ and 60DJ groups.

**Table 2:** Peak GRF, impulse, contact time and unit time force produced by upward and drop jumps.

	40DJ	60DJ	40UJ
	(n = 10)	(n = 10)	(n = 10)
Peak GRF (N*N^−1^)	9.94±0.46^‡^	14.03±0.54^‡ll^	6.30±0.31
Impulse (Nms)	1.099±0.047	1.163±0.092*	1.042±0.082
Contact time (ms)	0.110±0.011*	0.104±0.009^†^	0.127±0.011
Unit time force (N)	10.01±0.668^†^	11.21±1.278^†§^	8.23±0.343

All values are shown as means ± SD. 40DJ, rats that jumped downwards from a height of 40 cm; 60DJ, rats that jumped downwards from a height of 60 cm; 40UJ, rats that jumped upwards to a height of 40 cm. GRF, ground reaction force. Significant difference vs. 40UJ group: *p<0.05, ^†^ *p*<0.01, ^‡^ *p*<0.001.

Significant difference vs. 40DJ group: ^§^ *p*<0.05, ^ll^ *p*<0.001.

### Trabecular microstructure and mineralization

**Figure 3:**
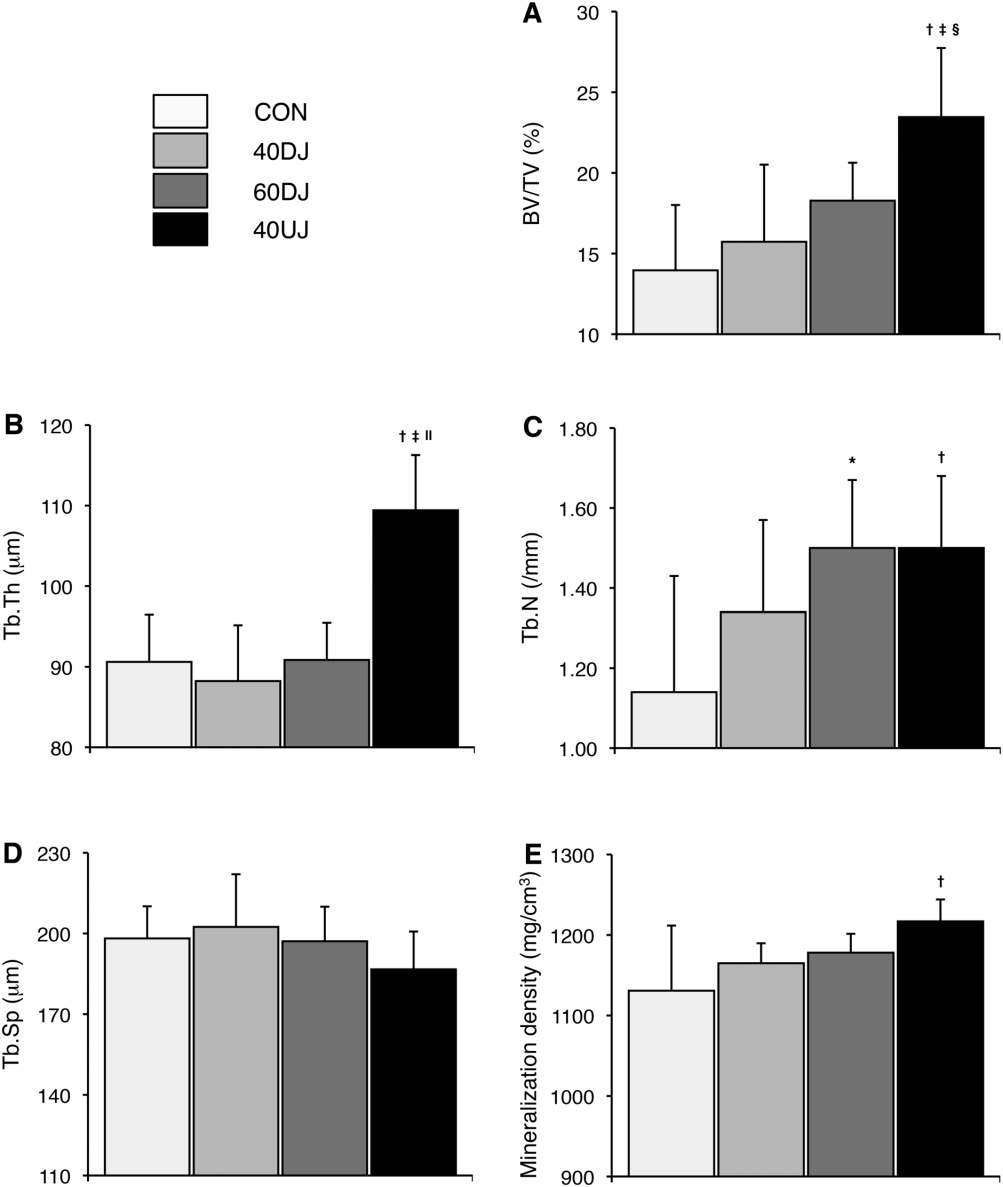
Microstructural parameters in the femoral metaphysis of rats that jumped downwards from a height of 40 (40DJ) or 60 (60DJ) cm, or upwards to a height of 40 cm (40UJ) or remained sedentary (CON) measured by micro-CT. A, Trabecular bone volume fraction (BV/TV); B, trabecular thickness (Tb.Th); C, trabecular number (Tb.N); D, trabecular separation (Tb.Sp); E, degree of trabecular bone mineralization. Significant differences: *p<0.01 and ^†^ *p*<0.001 vs. CON; ^‡^ *p*<0.001 vs. 40DJ; ^§^ *p*<0.05 and ^ll^ *p*<0.001 vs. 60DJ.

Figure 3 shows the 3D microstructural parameters in the distal femur. Among trabecular bone parameters, BV/TV and Tb.Th were significantly higher in the 40UJ, than the CON, 40DJ, and 60DJ groups and Tb.N was significantly higher in the 40UJ and 60DJ groups than in the CON group. However, Tb.Sp did not significantly differ among the four groups. The degree of trabecular bone mineralization was significantly higher in the 40UJ, than in the CON group.

### Histomorphometry

Table 3 shows the results of histomorphometric analysis of cancellous bone in the distal femur. Both BV/TV and Tb.Th were significantly higher in the 40UJ, than in the CON and 40DJ groups. With respect to bone resorption, both Oc.S/BS and ES/BS were lower in all jump groups than in the CON group, although the differences were statistically significant only in the 40DJ and 40UJ groups for Oc.S/BS and ES/BS, respectively. Values for Ob.S/BS and MS/BS reflecting bone formation were highest in the 40UJ group and significantly differed from those in the 60DJ group. In addition, MS/BS was also higher in the 40UJ, than in the CON group, whereas MAR and BFR/BS did not differ significantly among the four groups.

**Table 3:** Static and dynamic histomorphometry at distal femoral metaphysis of growing rats.

	CON	40DJ	60DJ	40UJ
	(n = 7)	(n = 7)	(n = 7)	(n = 7)
BV/TV (%)	17.7±5.0	16.4±2.3	19.1±4.8	24.7±5.0*^†^
Tb.Th (µm)	67.7±11.4	67.7±7.2	74.1±6.3	82.3±3.0*^†^
Tb.N (/mm)	2.6±0.5	2.4±0.2	2.6±0.4	3.0±0.6
Tb.Sp (µm)	330.7±84.7	348.7±39.1	327.1±78.6	262.9±76.9
Oc.S/BS (%)	15.5±2.1	11.2±2.3*	12.7±2.1	12.1±2.3
ES/BS (%)	21.3±2.6	17.5±3.3	19.6±4.3	15.9±2.1*
Ob.S/BS (%)	29.3±3.5	27.7±2.7	25.6±2.3	30.5±3.1^‡^
MAR (µm/day)	2.4±0.2	2.5±0.1	2.4±0.2	2.3±0.2
MS/BS (%)	34.7±4.9	35.3±4.0	31.3±1.6	41.1±3.6*^§^
BFR/BS (µm^3^/µm^2^/day)	0.81±0.1	0.89±0.1	0.75±0.1	0.97±0.1

Values are shown as means ± SD. n, number of rats in each group; 40DJ, rats that jumped downwards from a height of 40 cm; 60DJ, rats that jumped downwards from a height of 60 cm; 40UJ, rats that jumped upwards to a height of 40 cm. BFR/BS, bone formation rate/BS; BV/TV, trabecular bone volume fraction; CON, sedentary control group reared in breeding cage; MAR, mineral apposition rate; ES/BS, eroded surface/BS; MS/BS, mineralizing surface/BS; Ob.S/BS, osteoblast surface/BS; Oc.S/BS, osteoclast surface/bone surface; Tb.N, trabecular number; Tb.Sp, trabecular separation; Tb.Th, trabecular thickness. Significant difference vs. CON group: *p<0.05. Significant difference vs. 40DJ group: ^†^ *p*<0.05. Significant difference vs. 60DJ group: ^‡^ *p*<0.05; ^§^ *p*<0.0.

## Discussion

The main purpose of this study was to empirically test the hypothesis that a higher GRF produced during landing would be more effective for stimulating an elevation in trabecular bone mass compared with take-off. However, although landing was associated with a higher GRF, its ability to increase trabecular bone mass was weaker than that of take-off in this rat model of jump exercise. Our results showed that GRF alone cannot account for the impact of jumps on limb bone and suggest that local impact by muscle contraction serves an important role in increasing bone mass.

The effects of exercise on bone mass and strength depend on the loading magnitude, frequency and duration of stimulation, and the loading direction applied to the bone during the activity. High strain rates positively affect bone formation [18], [19]. Several published animal studies have found that jumping yields greater increases in bone mass and strength than running due to imposing greater anabolic stimulus upon the bone [1], [2], [3]. Human studies of high-impact activity have shown that exercise involving jumping elicits greater bone gain than repetitive high-frequency activities such as running or walking [20], [21]. Jumping impacts lower limb bones and is an essential athletic feature of many sports such as volleyball, basketball, gymnastics, triple jumping, dance, and figure skating. Ground reaction force is generated by the acceleration of all segments of a body that contact the ground. The actions of take-off and landing during jumps involve different magnitudes of GRF [22]. Reports indicate that the magnitude of the GRF during jumps in terms of athletic performance is greater during landing than during the take-off phase [5], [6]. Therefore, we postulated that the landing impact would result in more pronounced stimulation than the take-off impact. To the best of our knowledge, the effects of take-off and landing during jumps on bone mass and structure in rats as well as in humans has not been directly compared.

The effects of exercise on bone mass, strength, and structure of rodents have commonly been assessed using upward jumps [10], [11], [12], [13], [14], but also in rats dropped from specific heights [7], [8], [9]. One study has found that voluntary jump exercise including both upward jumps and landings produced significant increases in bone formation rates and BV/TV in cancellous bone of long-bone metaphyses [23]. It remains unclear, however, which phase of jumping is more effective for the increasing bone mass. Welch et al. [9] found that 10 free-fall landings per day produced significantly larger increases in the strength, geometry, and density of the forelimbs of growing rats without obvious changes in the hindlimbs. They also found that the cross-sectional area and breaking force were 20% and 40% higher at the ulnar diaphysis in free-fall, compared with control rats. Umemura et al. [1] found that upward jumps increased femoral cross-sectional area and breaking force by 14–15% and 13–18%, respectively. However, the present study found that bone mass and mineralization significantly increased only in the 40UJ, compared with the CON group. Drop jumps did not significantly improve trabecular bone mass except in Tb.N despite a higher GRF. These results suggested that trabecular bone mass at the distal femur is enhanced more effectively by forces generated on bone at take-off, than at landing. Wu et al. found similar results in female rhythmic sports gymnasts [24], in whom bone mass at the proximal femur was higher on the take-off (left) than the landing (right) leg. The apparent discrepancy between the results reported by Welch et al. [9] and the present findings might be explained by a difference in study design including the type of landing on the forelimbs instead of the hindlimbs. We designed a specific device to ensure that the rats landed on their hindlimbs during drop jumps to compare the effects of upward- and drop-jump impact at the same skeletal site.

Reports indicate that high-impact jumps with a GRF of 5–16 times body weight effectively increase bone mass in rats [9], [10], [14]. Here, the peak GRF was 9.94 and 14.03 times body weight in the 40DJ and 60DJ groups, respectively, which was higher than that of 6.30 in the 40UJ group. The range of peak GRF in the 60DJ group was similar to those of a previous study [9] that examined GRF during free-fall landing from a height of 60 cm. Drop jumps resulted in more than twice the peak GRF compared with upward jumps, but little affected trabecular bone mass, microarchitecture, and mineralization in the distal femur. The reason that drop jumps exerted less effects might be that GRF does not sufficiently reflect mechanical stress on trabecular bone at the femur. The notion that muscles and tendons absorb (dissipate) mechanical energy while eccentrically lengthening during the landing phase of jumps is generally accepted [25], [26], [27], [28]. Therefore, the transmission of GRF produced during landing might be attenuated by eccentric muscular action. On the other hand, during the take-off phase of jumps, stored elastic energy is used to enhance force production, which acts on the lower limb bone and could induce substantial local stress on bone. Both GRF as well as forces generated by muscular contraction should contribute to the loading of limb bones. Biomechanical analyses suggest that muscle contraction generates larger forces than body weight *per se* [29]. Moreover, the results of a human study *in vivo* by Lu et al. [30] showed that >70% of bending moments on a bone during gait are transmitted by force generated from muscle than from external reaction. Similarly, direct muscle contraction force on a bone during upward jumps might contribute to a substantial portion of the mechanical stress at the distal femur in addition to GRF.

Bone histomorphometry confirmed the positive effect of upward jumps on bone mass and dynamic histomorphometry showed that bone formation was stimulated more by upward, rather than drop jumps. Bone resorption parameters did not significantly differ between upward and drop jumps. Taken together, these results show that more osteogenic effects are generated by upward, rather than drop jumps.

One limitation of this study is that we did not measure the magnitude and rate of strain in femoral bone during the take-off and landing phases of jumps. Further studies are needed to define the mechanism(s) involved in skeletal responses to take-off and landing impact. Another limitation is that mechanical stress might be differently transmitted by muscle contraction at lower limb bones between humans and smaller animals, in which the relative contribution of muscle contraction to the impact on bone might be substantially higher. The specific effects of the upward phase of jumps on human bone have not yet been defined. Controlled studies of drop jumping in children have found positive effects on bone gain [20], [31]. Nonetheless, the present findings suggest that muscle contraction plays a key role in improving the trabecular bone mass and structure of limb bones.

### Conclusion

The present findings did not support the hypothesis that a higher GRF exerted by the landing phase would increase trabecular bone mass of the distal femoral metaphysis more than the take-off phase of jumps in growing rats. The results suggest that local impact accompanied by muscle contraction during jumps plays an important role in enhancing trabecular bone mass compared with impact expressed as GRF.
